# Histopathological Breast Cancer Image Classification by Deep Neural Network Techniques Guided by Local Clustering

**DOI:** 10.1155/2018/2362108

**Published:** 2018-03-07

**Authors:** Abdullah-Al Nahid, Mohamad Ali Mehrabi, Yinan Kong

**Affiliations:** School of Engineering, Macquarie University, Sydney, NSW 2109, Australia

## Abstract

Breast Cancer is a serious threat and one of the largest causes of death of women throughout the world. The identification of cancer largely depends on digital biomedical photography analysis such as histopathological images by doctors and physicians. Analyzing histopathological images is a nontrivial task, and decisions from investigation of these kinds of images always require specialised knowledge. However, Computer Aided Diagnosis (CAD) techniques can help the doctor make more reliable decisions. The state-of-the-art Deep Neural Network (DNN) has been recently introduced for biomedical image analysis. Normally each image contains structural and statistical information. This paper classifies a set of biomedical breast cancer images (BreakHis dataset) using novel DNN techniques guided by structural and statistical information derived from the images. Specifically a Convolutional Neural Network (CNN), a Long-Short-Term-Memory (LSTM), and a combination of CNN and LSTM are proposed for breast cancer image classification. Softmax and Support Vector Machine (SVM) layers have been used for the decision-making stage after extracting features utilising the proposed novel DNN models. In this experiment the best Accuracy value of 91.00% is achieved on the 200x dataset, the best Precision value 96.00% is achieved on the 40x dataset, and the best* F*-Measure value is achieved on both the 40x and 100x datasets.

## 1. Introduction

The unwanted growth of cells causes cancer which is a serious threat to humans. Statistics show that millions of people all over the world suffer various cancer diseases. As an example Table 1 summarises the statistics concerning the recent cancer situation in Australia. These statistics reveal the number of newly cancer-affected people diagnosed in Australia and also the number of people who died in 2017 in Australia. These statistics also divulge that the number of females affected and the number of females dying due to breast cancer are more than the numbers for males. This indicates that females are more vulnerable to breast cancer (BC) than males. Although these statistics are for Australia they might be representative of what is happening throughout the world.

Proper BC diagnosis can save thousands of women's lives, and proper diagnosis largely depends on identification of the cancer. Finding BC largely depends on capturing a photograph of the cancer-affected area which gives information about the current situation of the cancer. A few biomedical imaging techniques have been utilised, some of which are noninvasive such as Ultrasound imaging, X-ray imaging, and Computer Aided Tomography (CAT) imaging. Other imaging techniques are invasive such as histopathological images. Investigation of these kinds of images is always very challenging, especially in the case of histopathological imaging due to its complex nature. Histopathological image analysis is nontrivial, and the investigation of this kind of image always produces some contradictory decisions by doctors. Since doctors and physicians are human, it is natural that errors will occur.

A Computer Aided Diagnosis (CAD) system provides doctors and physicians with valuable information, for example, classification of the disease. Different research groups investigate opportunities to improve the CAD systems' performance. Some advanced engineering techniques have been utilised to take a general image classifier and adjust it as a biomedical image classifier, such as a breast image classifier. The state-of-the-art Deep Neural Network (DNN) techniques have been adapted for a BC image classifier to provide reliable solutions to patients and their doctors.

The basic working principle of DNN lies in the basic neural network (NN). Rosenblatt in 1957 [2] for the very first time introduced the NN concept, which provides decisions based on a threshold. Using some advanced engineering, a very light Convolutional Neural Network (CNN) model has been proposed by Fukushima [3], referred to as “Neocognitron.” The main interest of this project is to find stimulus patterns, where they can tolerate a limited amount of shifting variance. This “Negotron” model served as the first CNN model for biomedical signal analysis [3]. Specifically a CNN model has been for the first time introduced for breast image classification by Wu et al. [4] where they performed their experiments on a set of mammogram images. The utilisation of the CNN model for breast image classification has been limited due to its computational complexity, until Krizhevsky et al. [5] proposed their model known as AlexNet. This AlexNet model has brought about a revolutionary change in the image analysis field, specially image classification. Taking this model as a reference, a few other models have been adjusted such as ResNet [6], Inception [7], Inception-V4, and Inception-ResNet [8], for biomedical image classification. Jaffar classified the mammogram-image (MIAS-mini, DDSM) dataset using the CNN model and obtained 93.35% Accuracy and 93.00% Area Under the Curve (AUC) [9]. Qiu et al. [10] utilised a CNN for mammogram image classification where they utilised 2, 5, and 10 feature maps and obtained an average Accuracy of 71.40%. Ertosun and Rubin [11] employed the CNN method for automated positioning of the masses as well as breast image classification and obtained 85.00% Accuracy. Qui et al. [12] classified a set of mammogram images into benign and malignant classes, where they utilised a total of 560 Regions of Interest (ROI). Jiao et al. [13] characterised a set of mammogram images into benign and malignant images and obtained 96.70% Accuracy. A set of mammogram images has been classified by Sahiner et al., and their achieved ROC score is 0.87 [14]. M. M. Jadoon et al. classified a set of mammogram breast images into normal, benign, and malignant classes utilising a CNN model.

As with mammogram images, histopathological breast images have been classified by different research groups. Referring to the most recent, Zheng et al. classify a set of histopathological images into benign and malignant classes by locating the nucleus from the images using the blob detection method [15]. Araujo et al. classify a set of histopathological images utilising CNN into four classes (normal tissue, benign tissue, in situ carcinoma, and invasive carcinoma) and two classes (carcinoma and noncarcinoma). For the four-class classification they obtained 77.80% Accuracy, and when they performed the two-class classification they obtained 83.3% Accuracy [16]. Spanhol et al. utilised a CNN model and classified histopathological images from the BreakHis dataset containing four sets of images based on the magnification factor. They obtained a best image classification Accuracy of 85.6 ± 4.88% when they utilised the 40x magnification dataset [17].

**Figure 1:**
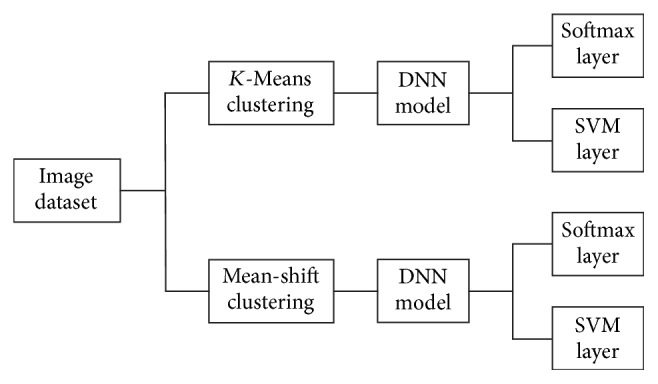
Overall image classifier model for benign and malignant image classification.

Images normally preserved a local as well as a hidden pattern which represent similar information. Histopathological images represent different observations of biopsy situation. The biopsy images which belong to the same groups normally preserve similar kinds of knowledge. Unsupervised learning can detect this kind of hidden pattern. The main contribution of this paper is to classify a set of biomedical breast cancer images using proposed novel DNN models guided by an unsupervised clustering method. Three novel DNN architectures are proposed based on a Convolutional Neural Network (CNN), a Long-Short-Term-Memory (LSTM), and a combination of the CNN and LSTM models. After the DNN model extracts the local and global features from the images the final classification decision is made by the classifier layer. As the classifier layer, this paper has utilised both the Softmax layer and a Support Vector Machine (SVM).  Figure 1 demonstrates the overall image classifier model which has been utilised in this experiment.

The remainder of this paper is organized as follows.  Section 2 describes the feature partitioning method based on clustering techniques.  Section 3 describes DNN models and this is followed by Section 4 which describes our proposed novel model based on the DNN method for the breast image classification.  Section 5 describes and makes a detailed analysis of the results.  Section 6 compares our findings with existing state-of-the art findings, and lastly Section 7 concludes the paper.

## 2. Feature Partitioning

Images naturally contain significant amounts of statistical and geometrical information. Representation of this kind of structural learning is a prior step for many data analysis procedures such as image classification. One of the techniques of finding the structural information is clustering the data in an unsupervised manner. Clustering allows the same kind of vector to be partitioned into the region. The clustering method partitions data of a similar nature and information in such a way that the partition between the grouped data is maximised. A few clustering methods are available. To find the hidden structure of the data, in this paper, we use the* K*-Means and Mean-Shift clustering algorithm approaches, which have been explained as follows:

- The* K*-Means (KM) algorithm is easy to implement, is less computationally complex, and can be calculated as shown in Algorithm 1.
- The Mean-Shift (MS) algorithm by nature is nonparametric and does not have any assumption about the number of clusters. The MS algorithm can be described as shown in Algorithm 2.

**Figure 2:**
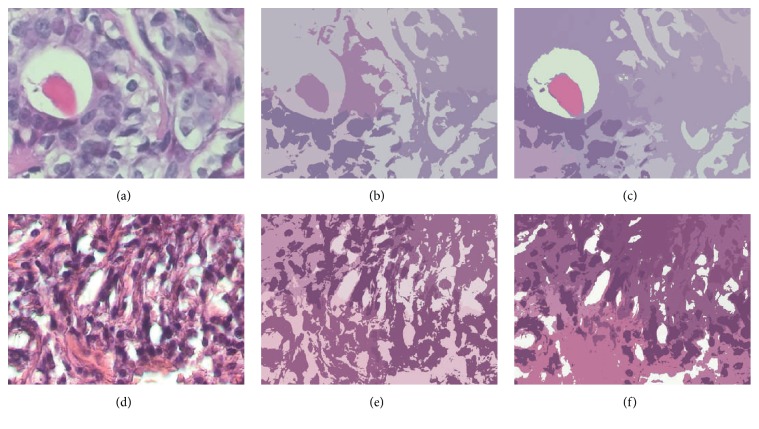
(a), (b), and (c) represent an original benign image, the KM cluster-transformed image, and the MS cluster-transformed image, respectively. (d), (e), and (f) represent an original malignant image, the KM cluster-transformed image, and the MS cluster-transformed image, respectively.

Figure 2 shows a benign and a malignant image and their clustering images.

## 3. Deep Neural Network

A Deep Neural Network is a state-of-the art technique for data analysis and classification. A few different DNN models are available, among them the Convolutional Neural Network (CNN) and Recurrent Neural Network (RNN). They have made some revolutionary improvements in the data analysis field. The following subsection will present the working principle of CNN and RNN (specially on the Long-Short-Term-Memory algorithm) and the working mechanism of the combination of the CNN and LSTM methods.

### 3.1. Convolutional Neural Network

A CNN model is an advanced engineering version of a conventional neural network where the convolution operation has been introduced, which allows the network to extract local as well as global features from the data, enhancing the decision-making procedure of the network. To perfectly control the workflow of a CNN network, along with a convolutional layer, a few intermediate layers have been introduced. These are explained in more detail below.

*Convolutional Layer. *A has been considered to be the main strength or key mechanism for the overall CNN model. In the convolutional layer the value of each position (*m*_1_, *m*_2_) of the input data *I*_*m*_1_×*m*_2__ has been convolved with the kernel *K*_*k*_1_×*k*_2__ to produce the feature map. The convolutional output of layer *l* and feature *t* for a particular data point (*m*_1_, *m*_2_) of the input data *I*_*m*_1_×*m*_2__ can be written as

(1) $$\begin{array}{c} I_{m_{1},m_{2}}⋆K_{k_{1}\times k_{2}}=\overset{\left(k_{1}-1\right)/2}{\underset{i=\left(-k_{1}+1\right)/2}{\sum }}\;\overset{\left(k_{2}-1\right)/2}{\underset{j=\left(-k_{2}+1\right)/2}{\sum }}I_{m_{1}-i,m_{2}-j}⋆K_{i\times j}. \end{array}$$

After adding the bias term *ℬ*^(*l*,*t*)^ the previous equation will be

(2) $$\begin{array}{c} F^{\left(l,t\right)}=\left(\;I_{m_{1},m_{2}}⋆K_{k_{1}\times k_{2}}\;\right)+B^{\left(l,t\right)}. \end{array}$$

Each of the neurons produces a linear output. When the output of a neuron is fed to another neuron, it eventually produces another linear output. To overcome this issue nonlinear activation functions such as

- Sigmoid
- TanH
- ReLU
- Leaky-ReLU

have been introduced.

**Figure 3:**
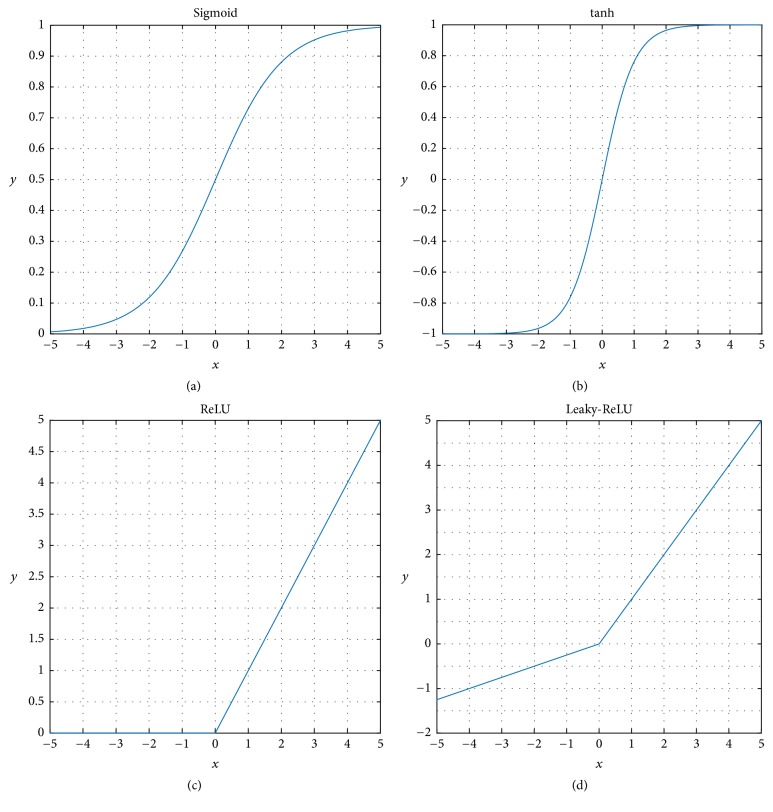
Sigmoid, TanH, ReLU, and Leaky-ReLU.

Figure 3(a) represents the Sigmoid function characteristic which follows the equation

(3) $$\begin{array}{c} \sigma \left(\;x\;\right)=\frac{1}{\left(1+e^{-x}\right)}. \end{array}$$

Interestingly this method suffers due to vanishing-gradient problems and having large computational complexity. Another nonlinear activation function is TanH which is basically a scaled version of the *σ*(*x*) operator such as

(4) $$\begin{array}{c} \tanh\left(\;x\;\right)=2\times \sigma \left(\;x\;\right)-1, \end{array}$$

which can avoid the vanishing-gradient problem and its characteristics are presented in Figure 3(b). The most popular nonlinear operator is Rectified Linear Unit (ReLU), which filters out all the negative information (like Figure 3(c)) and is represented by

(5) $$\begin{array}{c} ReLU\left(\;x\;\right)=\max\left(\;0,x\;\right). \end{array}$$

Figure 3(d) shows the Leaky-ReLU rectifier's characteristics, which is a modification of ReLU:

(6) $$\begin{array}{c} Leaky-ReLU\left(\;x\;\right)=\sigma \left(\;x\;\right)+\beta ReLU\left(\;x\;\right), \end{array}$$

where *β* is a predetermined parameter.

The main ingredient of the convolutional layer is the kernel, which scans through all the input data and tries to extract the global features. The number of steps a kernel takes each time is known as the stride. The border row and column positions might not be convolved perfectly if we select imperfect stride steps and size. To perfectly conduct the convolution operation at the border, a few extra rows and columns (with all zeros) are added, which is known as zero padding.

**Figure 4:**
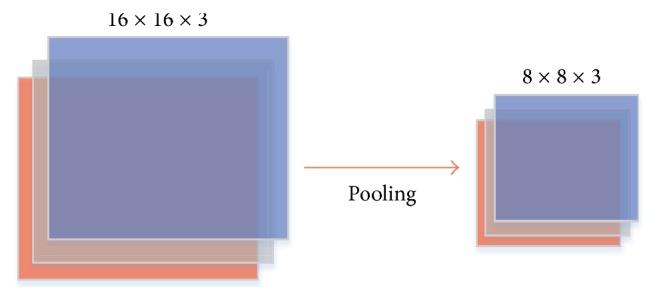
Pooling operation performed by 2 × 2 kernel.

The convolutional model produces a significant amount of feature information. As the model structure increases, the amount of feature information also increases, which actually increases the computational complexity and makes the model more sensitive. To overcome this kind of problem, a sampling process has been introduced:

- *Subsampling*: subsampling or pooling is the procedure known as downsampling the features to reduce dimensionality. Eventually it reduces the overall dimensionality and complexity. Four types of pooling operation are available:
   (a) Max-Pooling
   (b) Average Pooling
   (c) Mixed max-average pooling
   (d) Gated max-average pooling.
- Figure 4 illustrates a generalised pooling mechanism for a CNN model.

A DNN deals with a large number of neurons, which enables the network to take a direction where the network takes into consideration a large number of predictions. This kind of situation provides very good performance in the training dataset and worse performance for the test dataset. This kind of problem is known as an overfitting problem. To overcome this kind of problem the drop-out procedure has been introduced. It is described in more detail below.

**Figure 5:**
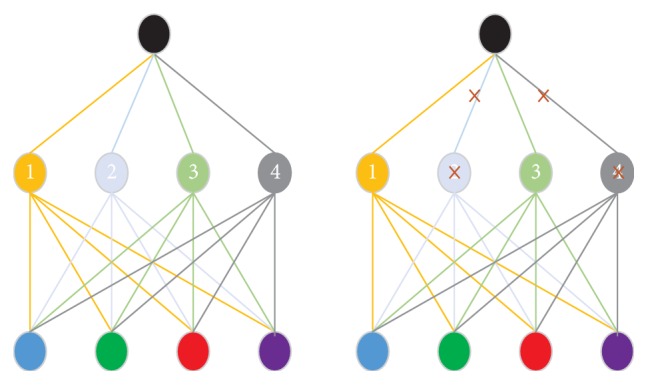
Drop-out.

- *Drop-out*: some of the neurons are randomly removed to overcome the overfitting problem. In this procedure a few of the neurons are randomly dropped out (with some predefined probability) so that the network can learn more robust features.  Figure 5 shows a simplified example of a drop-out mechanism. The right-hand side image shows that the network contains four hidden neurons 1 to 4; in the left-side image neurons 2 and 4 have been removed so that these two neurons do not have any effect on the network decision.

At the end of the network, all the neurons are arranged in a flattened way. The neurons of the flat layer are fully connected to the next layer and behave like a conventional neural network. Normally more than one fully connected layer is introduced. Consider the last layer as the “end” layer; then, at the layer before the “end” layer, there must be at least one flat layer or fully connected layer. Then the end layer function can be represented as

(7) $$\begin{array}{c} F_{k}^{end}=\overset{end-1}{\underset{j=1}{\sum }}w_{k,j}^{end}F_{g}^{end-1}+B_{k}^{end-1}. \end{array}$$

**Figure 6:**
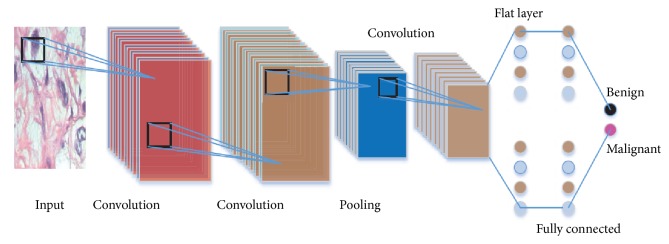
Workflow of a Convolutional Neural Network.

Figure 6 depicts a generalised CNN model for image classification. The end layer can be considered as the decision layer.

#### 3.1.1. Decision Layer

In the decision layer Softmax-Regression techniques as well as the Support Vector technique are utilised.

(i) In the Softmax layer, the cross-entropy losses are calculated such as
  (8) $$\begin{array}{c} L_{k}=-\ln\left(\;{\bar{y}}_{k}\;\right), \end{array}$$
where ${\bar{y}}_{k}$ can be written as
  (9) $$\begin{array}{c} {\bar{y}}_{k}=\frac{\exp\left(F_{k}^{end}\right)}{\sum_{k=1}^{2}\exp\left(F_{k}^{end}\right)}. \end{array}$$
Here *k* = {1,2} where 1 is for benign and 2 is for malignant case. The value of *L*_*k*_ provides the final decision such as if *L*_1_ > *L*_2_ the network will produce malignant output.
(ii) *Support Vector Machine*: instead of a Softmax layer, an SVM [20] layer can be used including the following conditions. For a generalised case, let *x* = *x*_1_, *x*_2_,…, *x*_*n*_ be the training data and *y* = *y*_1_, *y*_2_,…, *y*_*n*_ be the corresponding label. If we consider that the data is linearly separable then the optimisation constraint is considered as *yW*^*T*^*x* ≥ 0. However, sometimes data is not linearly separable; in that case soft thresholding has been introduced and the constraint redefined as *yW*^*T*^*x* ≥ 1 − *ξ*_*i*_, where *ξ*_*i*_ = 0. Now the optimisation problem is redefined as
  (10) minw,ξi 12WTW+C∑ξis.t. ξi≥1−yiWTWxi,ξi≥0  ∀i.

### 3.2. LSTM

**Figure 7:**
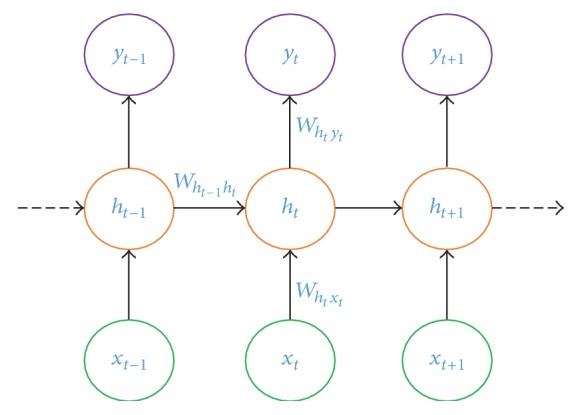
A generalised RNN model, where the RNN output is computed and the reference information passes through the hidden unit.

While a CNN learns from scratch, an error signal is fed back to the input. In a Recurrent Neural Network, instead of learning from scratch the network learns from the reference point. The output of a particular layer is fed back to the input which works as the reference input. A generalised RNN model is presented in Figure 7. Let the sequence of input vectors be **X** = {*x*_1_, *x*_2_,…, *x*_*R*_}, the hidden state be **H** = {*h*_1_, *h*_2_,…, *h*_*H*_}, and the output state be **Y** = {*y*_1_, *y*_2_,…, *y*_*o*_}, where

(11) $$\begin{array}{c} y_{t}=\sigma \left(\;W_{h_{t}y_{t}}h_{t}+b_{t}\;\right). \end{array}$$

Here, *W*_*h*_*t*_*y*_ represents the weight vector from the hidden unit *h*_*t*_ to the output unit *y*_*t*_ for the sequence *t*, where *h*_*t*_ is defined as

(12) $$\begin{array}{c} h_{t}=\sigma \left(\;W_{h_{t-1}h_{t}}h_{t-1}+W_{x_{t}h_{t}}x_{t}+b_{h_{t}}\;\right). \end{array}$$

Here, *h*_*t*−1_ represents the output of the hidden unit for the sequence *t* − 1; *W*_*h*_*t*−1_*h*_*t*__ represents the weight vector from the hidden unit *h*_*t*−1_ to the hidden unit *h*_*t*_ for the sequence *t*; *b*_*h*_*t*__ represents the bias; *W*_*x*_*t*_*h*_*t*__ represents the weight vector from the input sequence *i*_*t*_ to the hidden unit *h*_*t*_.

**Figure 8:**
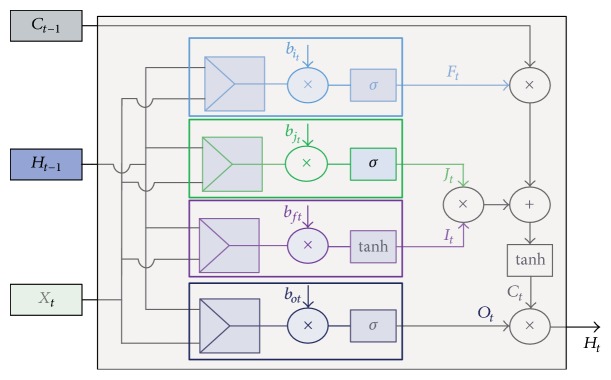
A generalised cell structure of an LSTM.

A normal RNN suffers due to a vanishing-gradient probability. To overcome this problem, the Long-Short-Term-Memory (LSTM) architecture has been introduced by Hochreiter and Schmidhuber [21]. One notable feature of the LSTM method is that it contains the “for gate” through which the network controls the flow of information.  Figure 8 represents the cell structure of an LSTM network. The main parameters of the LSTM network can be represented as

(13) it=tanh⁡Wxtitxt+Wht−1itht−1+bit,jt=σWxtjtxt+Wht−1jtht−1+bjt,ft=σWxtftxt+Wht−1ftht−1+bft,ot=σWxtotxt+Wht−1otht−1+bot,ct=ct−1⊙ft+it⊙jt,ht=tanh⁡ct⊙ot.

*f*_*t*_ is the forget gate, *i*_*t*_ is the input gate, *h*_*t*_ provides the output information, and *c*_*t*_ represents the cell state [22]. Here the weight matrix and bias vectors are **W**_××_ and **b**_×_.

### 3.3. CNN-LSTM

A CNN has the benefit of extracting global information. On the other hand, an LSTM has the ability to take advantage of long-term dependencies of the data sequences. To utilise both these advantages, the CNN and LSTM models have been hybridised together for the classification [23–25].

**Figure 9:**
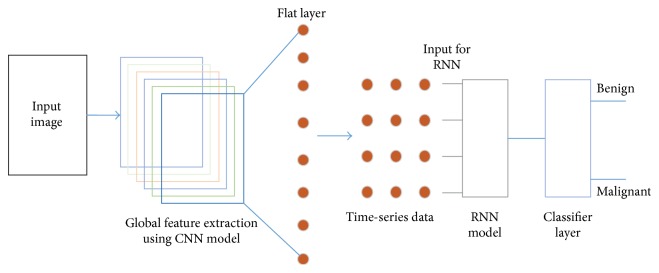
CNN and LSTM models combined.

From the output of the CNN model, it is difficult to generate an undirected graph to make the data into the time-series format, so that the network can extract the dependencies of the data. To do this we have converted the convolutional output (which is 2-dimensional) into 1D data.  Figure 9 represents the basic structure of the LSTM and CNN model.

## 4. Proposed Model

**Figure 10:**
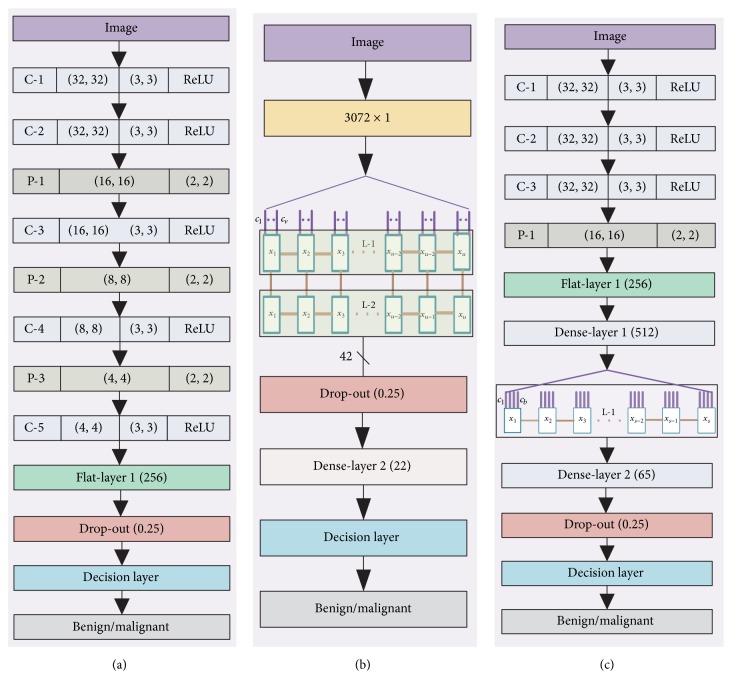
Conventional CNN, LSTM based architecture (a, b), and CNN-LSTM based architecture (c).

We have utilised three different models for our data analysis (Figure 10). Model 1 utilises CNN techniques, and Model 2 utilises the LSTM structure, whereas Model 3 employees both the CNN and LSTM structures together for the data analysis.

### 4.1. Model 1

In this method, the input image is convolved by a 3 × 3 kernel, and the output of each kernel is passed through an ReLU activation filter in layer C-1. Each kernel strides one step each time, and to keep the border information intact, we have added two extra rows and columns with a value of “0.” This ensures that the newly created feature maps are also 32 × 32 in size. After the C-1 layer another convolutional layer named C-2 has been introduced, with the same kernel size 3 × 3 and an ReLU rectifier.

After the C-2 layer the pooling operation P-1 is performed with the kernel size 2 × 2. As we have utilised a 2 × 2 kernel size, each of the feature maps decreases in size from 32 × 32 to 16 × 16. After the P-1 layer another convolutional layer called C-3 has been utilised, with an ReLU rectifier. Each of the feature maps of the C-3 layer was 16 × 16; due to utilising the P-2 (pooling layer of 2 × 2 kernel) layer the feature map is now 8 × 8. After the C-4 layer another pooling operation has been performed named P-3 followed by a convolutional layer C-5. The output of the convolutional layer has been flattened. The C-5 layer contains 16 feature maps and each of the feature maps is 4 × 4 in size, so the flattened layer contains 256 features. Twenty-five percent of the information has been dropped out in the drop-out layer before sending them through the decision layer (SVM/Softmax) to provide the benign or malignant decision.

### 4.2. Model 2

In the second model we utilised the LSTM method, which is a branch of the RNN model. Our input image is in two-dimensional format. To make it a suitable format for the LSTM model we have converted the data to 1D data format, and the newly created data vector is 3072 × 1 in size, as our input data is 32 × 32 × 3. The one-dimensional data has been converted to time-series data. To fit the 3072 × 1 into time-series data, we have created Time Steps (TS) data *x*_1_ to *x*_*u*_ and the Input Dimension of each of the TS is a *v* such as *c*_1_ to *c*_*v*_, where *v* × *u* = 3072. We stacked two LSTM layers consecutively, specifically L-1 and L-2. The output of the LSTM layer L-2 produces 42 neurons. The output of the LSTM layer is passed through the drop-out layer with a 25% probability. After the drop-out layer a dense layer has been introduced which contains 22 neurons. Finally a decision layer has been utilised to make the decisions about benign and malignant classes.

### 4.3. Model 3

In this model we have utilised both the CNN model and the LSTM model together. At first the input image is convolved by the convolutional layer C-1 with a 3 × 3 kernel along with a ReLU rectifier. This layer produces feature vectors and the size of each feature vectors is 32 × 32. Consecutively there are another two layers, C-2 and C-3, placed one after another. After the layer C-3 one pooling layer named P-1 has been introduced with the kernel size 2 × 2. As the pooling layer uses a 2 × 2 kernel, the output of P-1 produces a 16 × 16 kernel. After the P-1 layer a flat layer has been introduced, followed by a dense layer which produces 512 neurons. The output of this layer has been used as the input layer for the LSTM. As this layer contains a one-dimensional vector, we have converted this data into a time series. We have created TS data *x*_1_ to *x*_*s*_ and each of the TS data has contained an ID of size *q* such as *c*_1_ to *c*_*b*_ where *s* × *b* = 512. After the LSTM layer one dense layer of 65 neurons has been placed followed by a drop-out of 25% of the data. After that a decision layer has been placed which distinguishes the benign and malignant data.

## 5. Results and Discussion

**Figure 11:**
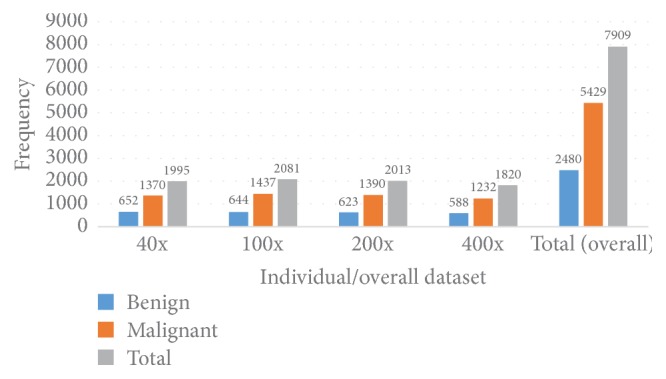
Statistical breakdown of the BreakHis dataset.

We have utilised the BreakHis breast image dataset for our experiment [17]. All the images of this dataset have been collected from 82 patients and the sample collection has been performed in the P&D Laboratory, Brazil. This dataset contains four groups of images depending on the magnification factor 40x, 100x, 200x, and 400x. Each of the images of this dataset are RGB in nature and 760 × 460 pixels in size and they are elements of a particular set {Benign, Malignant}.  Figure 11 shows the group-wise statistics as well as the overall statistics of this dataset.

As Figure 11 shows, there are 7909 images where 2480 are benign and the rest are malignant, which indicates that almost 70.00% of the data are malignant. For an individual magnification case, that is, if we consider 40x, 100x, 200x, and 400x individually, in all the cases almost 70.00% of the data are malignant. This shows that this dataset is imbalanced; more specifically, this dataset is more biased towards malignant in terms of frequency.

### 5.1. Performance of a Different Model

Following subsections analyze the performance of the algorithms based on parameters such as True Positive (TP/Sensitivity), False Positive (FP), True Negative (TN/Specificity), False Negative (FN), Accuracy, Precision, recall, and Matthews Correlation Coefficient (MCC). For the sake of comparison we have also performed all the experiments on the original images and this particular case is represented as (OI). When we utilised the KM algorithm we have fixed the cluster size (*K*) to 8, and when we utilised MS algorithm we have fixed the Bandwidth (BW) at 0.2.

#### 5.1.1. TP/FP/TN/FN Performance

This subsection describes the True Positive (TP/Sensitivity), False Positive (FP), True Negative (TN/Specificity), and False Negative (FN) performance from this experiment, and the data related to this experiment are presented in Table 2.

For the 40× dataset the best True Positive (TP) value (95.00%) is achieved when Model 3 is utilised along with the MS cluster algorithm and the SVM classifier together. Model 2 also provides the same kind of TP value, 94.76%, when the MS and SVM algorithms are utilised together. In this particular case the TN values for Model 3 and Model 2 are 59.10% and 53.55%, respectively. However, when Model 1 is utilised in this particular scenario the TN value is 68.39% and the FP value is 31.60%. For the 40x dataset, the best TN value is achieved when the MS cluster method and Softmax decision algorithm are utilised, and in this particular case the TP value is 81.00% for Model 1. When the original image (OI) is utilised, of the three models, Model 1 provides the best TN and TP values, 78.00% and 94.00%, respectively. In this particular case a Softmax decision layer has been employed.

For the 100x dataset the best TP value achieved 95.96% when we use KM clustering techniques and the Softmax decision algorithm together. In this particular case the TN value is 75.00% and the FP value is 25.00%. The best TN value, 80.20%, is achieved when we utilised the MS clustering algorithm and the Softmax algorithm together, and in this particular case the FP value is 19.80%. When the original image (OI) is utilised, the best TP value 93.00% is achieved when Model 3 along with the SVM decision algorithm has been applied.

When we use the 200x dataset the best TP value, that is, 97.00%, is achieved when the MS clustering algorithm and the Softmax layer are utilised. However in this case the TN value is 65.00% and the FP value is 35.00%. When we use Model 1, MS, and SVM classifier together for the 200x dataset, the TP value is 95.80% and in this case the TN and FP values are 70.70% and 29.00%, respectively. For the 200x dataset the best TN value, 81.00%, is achieved when the MS and Softmax algorithms are utilised with Model 1, and in that particular case the FP value is 19.00%, the TP is 96.00% and the FN value is 3.60%, respectively. A 96.00% TP value is achieved when the original image is utilised along with Model 1. In this particular case the SVM decision algorithm has been used.

For the 400x dataset the best TP value achieved is 96.00% when KM and the Softmax layer along with Model 1 are utilised together. The best TP value achieved is 95.31% when we utilised Model 1 and the MS and SVM algorithms together. In this particular case the TN and TP values are 68.30% and 31.69%, respectively. When we utilised the 400x dataset the best TN value 84.00% is achieved when we use the MS and Softmax algorithms (for Model 1) and the subsequent TP value is 93.00%. A 94.40% TP value is achieved when the original image is utilised along with Model 1 and the SVM Decision Algorithm. The best TP value is achieved when the original image is utilised along with Model 3 and the Softmax Decision Algorithm.

#### 5.1.2. Accuracy Performance

**Figure 12:**
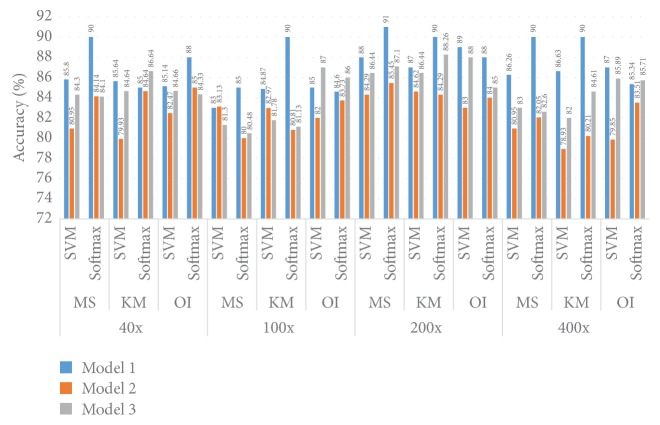
Comparison of the Accuracy in Model 1, Model 2, and Model 3.

Figure 12 illustrates the Accuracy information for different models and different datasets. For the 40x dataset the best Accuracy achieved is 90.00% when Model 1, the MS clustering method, and a Softmax layer are utilised together. For the 40x dataset and SVM classifier together, irrespective of the MS and KM clustering method, the Accuracy performance is almost the same at 86.00%. For the 40x dataset, of all three models, Model 1 gives the best performance for all cases irrespective of the cluster method as well as the classifier method. When we use the 40x dataset the best Accuracy performance is achieved when Model 1 and a Softmax layer are combined.

For the 100x dataset and the MS cluster method along with the SVM method, Model 2 provides the best performance, 83.13%, and this same kind of Accuracy performance, 83.00%, is shown by Model 1. When the KM cluster and SVM classifier are used together, Model 1 provides 84.87% Accuracy followed by Model 2 (82.97%) and Model 3 (81.78%). When the Softmax classifier is utilised Model 1 elicits the best Accuracy performance irrespective of the clustering method, whether the MS or KM cluster method is employed. Model 1 and Model 3 provide the same kind of Accuracy performance of around 81.00% when we use the Softmax classifier, and this result remains the same whether we use the MS or KM cluster algorithm. When we use original images, of the three models, Model 3 provides the best Accuracy performance, 87.00%, where SVM classifier layers have been utilised.

When we use the 200x dataset and the MS clustering algorithm, for all the models the Softmax classifier performs better than the SVM classifier. The best Accuracy of 91.00% is achieved when we use Model 1. For the* K*-M cluster algorithm, the Softmax classifier provides better performance than the SVM classifier. When we use the original images the best Accuracy is achieved when Model 1 has been utilised along with an SVM classifier layer.

For the 400x dataset with the Softmax classifier, the best Accuracy performance (90.00%) is achieved when we utilised Model 1 irrespective of the MS or KM algorithm. When we utilised the SVM algorithm Model 1 provides better Accuracy (around 82.26%) than Model 2 and Model 3. For the 400x dataset Model 1 shows the best performance when we utilised an SVM layer.

#### 5.1.3. Precision Performance

**Figure 13:**
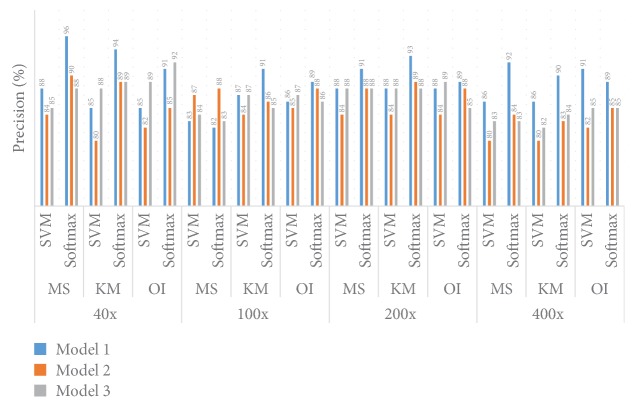
Comparison of Precision between Model 1, Model 2, and Model 3.

Figure 13 shows the Precision information for different models and different datasets. For the 40x dataset the best Precision performance (96.00%) is achieved when the MS cluster algorithm and a Softmax layer are utilised with Model 1. When the KM clustering algorithm and Softmax classifier are utilised together, the best Precision (94.00%) is achieved when we employed Model 1. Interestingly, when the KM clustering method and Softmax layer are utilised both Model 2 and Model 3 give a similar Precision of 89.00%. The worst Precision value (80.00%) is achieved when we utilise the KM clustering algorithm and SVM classifier with Model 2. Overall, for the 40x dataset, the SVM classifier provides the worst performance when the Softmax layer is utilised. When we utilise original images the best Precision value (92.00%) is achieved for Model 3 along with a Softmax decision layer.

For the 100x dataset the best Precision (91.00%) is achieved when we use the KM clustering algorithm along with the Softmax layer with Model 1. In this particular situation, Model 2 and Model 3 provide 86.00% and 85.00% Precision, respectively. For KM clustering and SVM classifier both Model 1 and Model 2 achieve 87.00% Precision. When the MS clustering method is implemented the best performance is achieved when Model 3 is used along with the Softmax layer. For the MS clustering method, Model 1 and Model 3 provide similar levels of Precision. When we utilise original images the best Precision value (89.00%) is achieved for Model 1 along with a Softmax decision layer.

For the 200x dataset the best Precision (93%) is achieved when the KM clustering method and a Softmax layer and Model 1 algorithm are utilised together. In this particular case Model 2 and Model 3 provide a similar Precision of 89.00% and 88.00%, respectively. For the MS cluttering algorithm, the best Precision, 91.00%, is achieved when Model 1 is utilised. For the KM clustering algorithm and the SVM method the Precision achieved is 88.00%. When we utilise original images the best Precision value (89.00%) is achieved for Model 1, and this result is true for the SVM as well as the Softmax decision layer.

For the 400x dataset, the best performance is achieved when the MS clustering method along with the Softmax layer is utilised; Model 1 provides the best Precision (92.00%). In this particular case Model 2 and Model 3 provide 84.00% and 83.00% Precision, respectively. With the KM clustering and the Softmax layer together the Precision value is 90.00%. Overall, the Softmax layer provides the best Precision values. A 91.00% Precision value is achieved for Model 1 and the SVM Decision layer algorithm when an original image has been provided as input.

#### 5.1.4. *F*-Measure Performance

**Figure 14:**
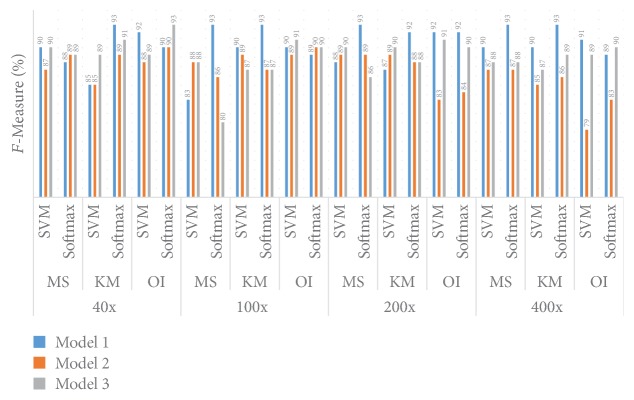
Comparison of* F*-Measure between Model 1, Model 2, and Model 3.

Figure 14 shows the* F*-Measure information for different models and different datasets. For the 40x dataset when the KM clustering method with the Softmax layer is used, an* F*-Measure 93.00% value is achieved when Model 1 is utilised. In that particular scenario Model 2 gives a 91.00%* F*-Measure and Model 3 an 89.00%* F*-Measure. For the MS clustering algorithm and SVM classifier algorithm, Model 1 and Model 2 provide 90.00%* F*-Measure values. In this particular clustering algorithm, when the Softmax layer is employed all the models provide the same performance, around 89.00%. A 93.00%* F*-Measure value is achieved when we utilise Model 3 along with the Softmax algorithm and original image.

For the 100x dataset Model 1 provides the best* F*-Measure of around 93.00% when the Softmax layer algorithm is employed; this performance is true for both the MS and KM clustering methods. When KM clustering and the Softmax layer are combined together Model 2 and Model 3 provide the same* F*-Measure of 87.00%. When the KM clustering method is utilised with the SVM classifier, Model 1 gives a 90.00%* F*-Measure while Model 2 and Model 3 provide 89.00% and 87.00%* F*-Measure values, respectively. When the MS clustering algorithm is combined together with Model 2 and Model 3 they provide the same* F*-Measure of 88.00%, and with this particular scenario Model 1 provides an 83.00%* F*-Measure. A 91.00%* F*-Measure value is achieved when we utilise Model 1 along with the SVM algorithm at the decision layer and provide original image. In this particular case when we utilise the Softmax layer both Model 2 and Model 3 provide similar* F*-Measure values.

For the 200x dataset, the best* F*-Measure of 93.00% is provided by Model 1 when the MS algorithm and Softmax layer are combined. However, when the KM cluster is utilised along with the Softmax layer the* F*-Measure is 92.00%. In this particular scenario, both Model 2 and Model 3 provide a similar* F*-Measure value of 88.00%. When KM clustering and the SVM algorithm are utilised together Model 3 provides a 90.00%* F*-Measure, Model 2 provides an 89.00%* F*-Measure, and in this particular case Model 1 provides an 87.00%* F*-Measure. When SVM and the Softmax layer are used together Model 1, Model 2, and Model 3 provide 88.00%, 89.00%, and 90.00%* F*-Measure, respectively. A 92.00%* F*-Measure value is achieved when we utilise Model 1 and original image, and this is true for both the Softmax and SVM algorithms.

For the 400x dataset Model 1 provides the best* F*-Measure value of 93.00% irrespective of the clustering method. For the KM clustering algorithm and SVM algorithm, the* F*-Measure values are 90.00%, 85.00%, and 87.00% for Model 1, Model 2, and Model 3, respectively. When the MS clustering method and SVM algorithm are utilised together Model 1, Model 2, and Model 3 provide 90.00%, 87.00%, and 88.00%* F*-Measure values, respectively.

#### 5.1.5. Accuracy, Loss, and MCC Performance at Different Epochs

**Figure 15:**
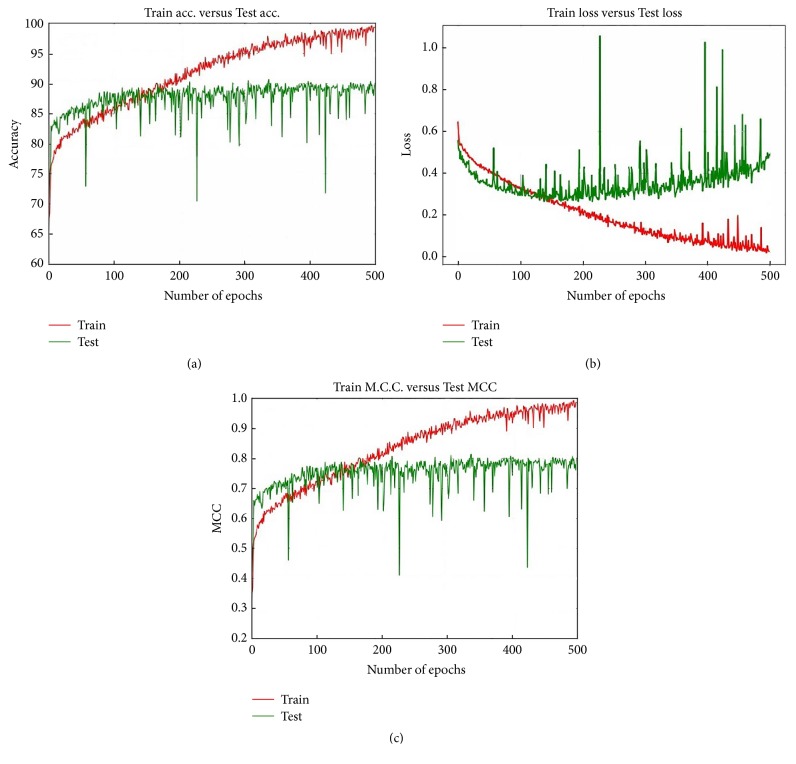
Accuracy Loss and MCC values for Model 1 when we have utilised 40x dataset MS and Softmax together.

The best Accuracy performance is achieved when we utilised Model 1 along with MS clustering and the Softmax layer on the 40x dataset. Figures 15(a), 15(b), and 15(c) represent, respectively, the Accuracy, loss, and MCC values for this particular situation. Initially the Test Accuracy shows better performance than the Train Accuracy. Up to around epoch 180, the Train Accuracy is better than the Test Accuracy. After the epoch 180 the Train Accuracy exhibits superior performance than the Test Accuracy.

After epoch 300 the Train Accuracy remains constant at about 90.00%. Interestingly, after around epoch 180 the Train Accuracy outperforms the Test Accuracy; after around epoch 180 the difference in Accuracy performance between the Train and Test increased, with the Test remaining constant.

**Figure 16:**
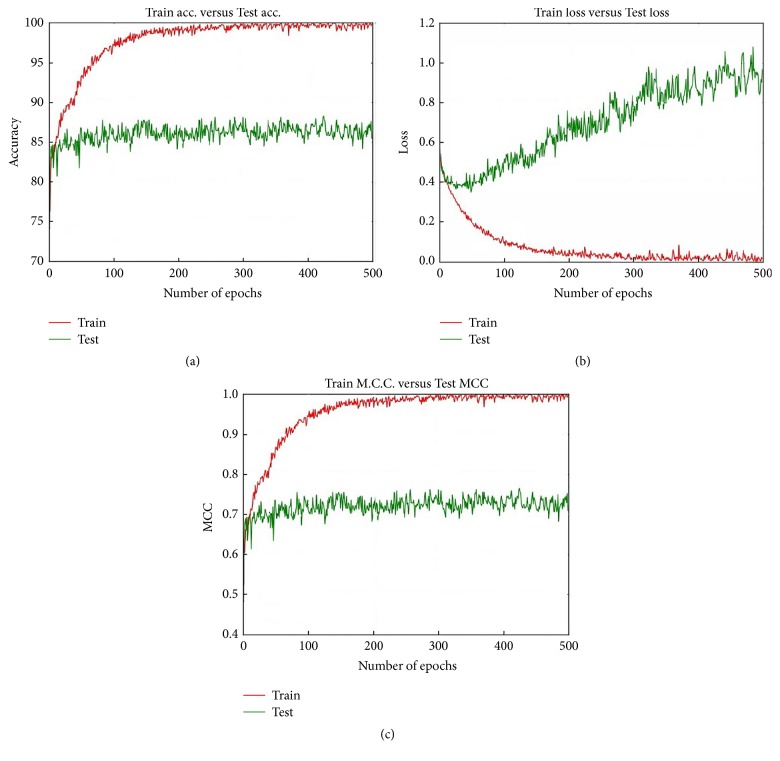
Accuracy, loss, and MCC values for Model 2 when we utilise the 200x dataset, MS, and Softmax together.

Model 2 provides the best Accuracy with the 200x dataset and the MS algorithm and Softmax layer.  Figure 16 shows the Accuracy, loss, and MCC values for this particular case for epoch 500. On virtually every occasion the Train Accuracy performance is better than that of the Test Accuracy. After about epoch 100 the Test Accuracy almost remained constant; however the Train Accuracy continuously increased, and after epoch 300 the Train Accuracy reaches 100% and remains constant throughout the epochs.  Figure 16(b) shows that the Train loss continuously decreases and the Test Accuracy steadily increases. As the epoch progresses the gap between the train loss and test loss continuously increases. The test MCC remains almost constant around 0.73 while the train MCC value continuously increases and reaches 1 and remains constant.

**Figure 17:**
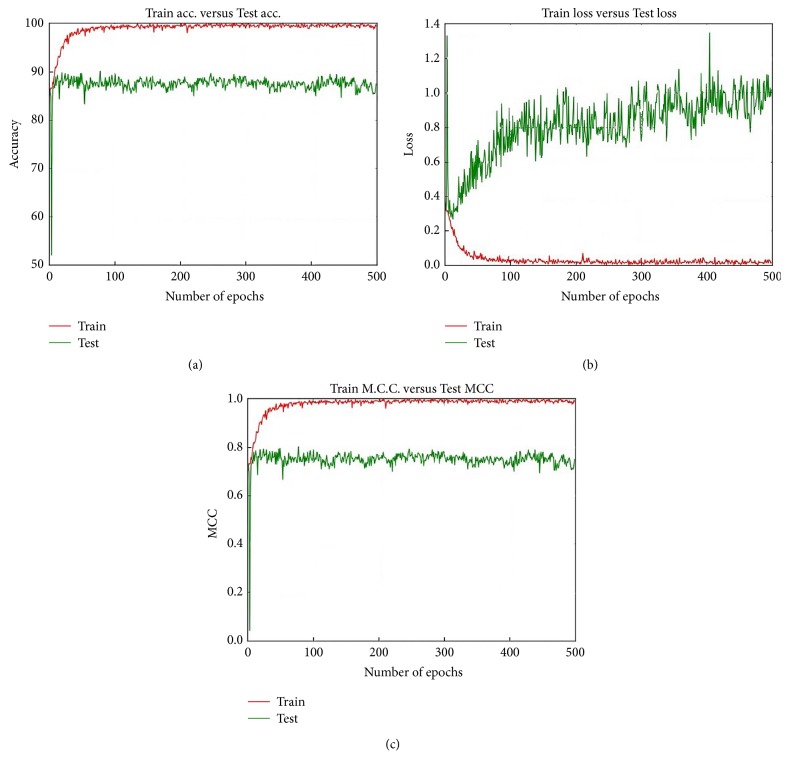
Accuracy, loss, and MCC values for Model 3 with the 200x dataset, KM, and Softmax together.

Model 3 is the most accurate with the 200x dataset and the KM and Softmax layer.  Figure 17 shows the Accuracy, loss, and MCC values for this particular case for epoch 500.  Figure 17(a) shows that the Train Accuracy is almost always higher than the Test Accuracy. The difference between the Train Accuracy and the Test Accuracy increases with the epoch up to around epoch 100. After epoch 100 the Test Accuracy remains constant at around 88.00% and the Train Accuracy remains constant at 100.00%. For the loss performance, the Test loss reduces as the epoch progresses on and the Train loss value remains virtually constant. The MCC value for the test (around 78.00%) remained constant after around epoch 20. The train MCC value reached the highest value, of 1.00, after around epoch 100.

### 5.2. Effect of TS and ID

TS and ID have an effect on LSTM performance. In this subsection we analyze the effect of the TS and ID values with reference to Accuracy, average time, and required parameters for Model 2.

Table 3 summarises the average time and parameters required for Model 2 performance with different combinations of TS and ID. When TS and ID are fixed at 24 and 128, respectively, the required average time is 191 seconds and a total of 5808 parameters are required. This table also exhibits a very interesting behaviour. As we increase the value of TS and reduce the value of ID, the number of required parameters to execute the CNN model has fallen. However, the time required to execute the model increased.

**Figure 18:**
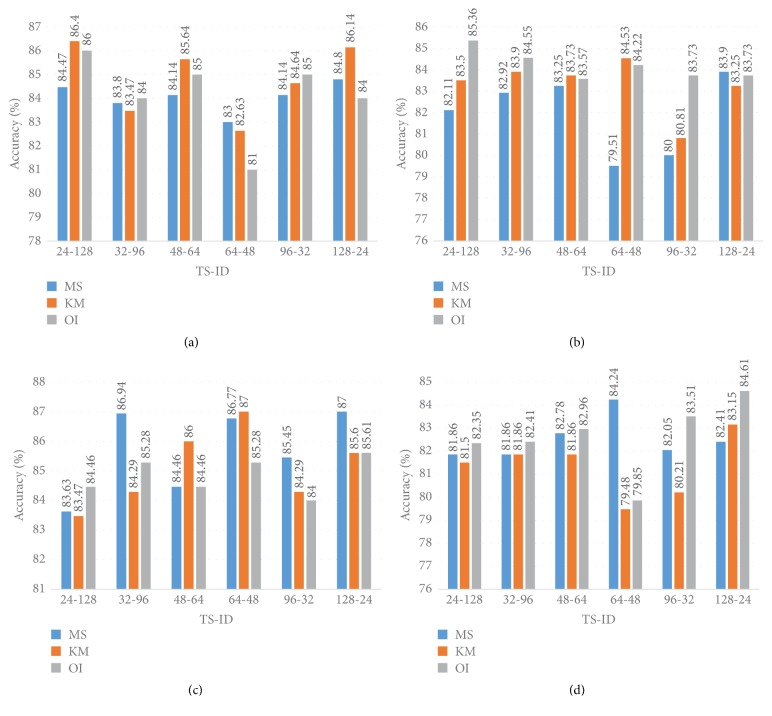
(a), (b), (c), and (d) represent the Accuracy for the 40x, 100x, 200x, and 400x datasets for Model 2 with varying TS and ID.

For the 40x dataset, Figure 18(a) shows the Accuracy where the TS and ID values have been varied. When TS and ID are fixed at 24 and 128, respectively, the obtained Accuracy for the MS, KM, and OI methods were 84.47%, 86.4%, and 86.00%, respectively.  Figure 18(b) displays the Accuracy performance on the 100x dataset with different TS and ID values. Where TS = 24 and ID = 128, 85.36% Accuracy is achieved when the original image is utilised. When the TS value is fixed at 128 and ID is fixed at 24, the MS method provides Accuracy at 83.90%. For the 200x dataset, 86.94% Accuracy has been achieved using the MS method with the TS and ID values 64 and 96, respectively. When TS and ID are fixed at 128 and 24, respectively, the Accuracy was 87.00%. For the 400x dataset 84.24% Accuracy is achieved when the MS method is utilised, where TS is fixed at 64 and ID is fixed at 48.

### 5.3. The Effect of Cluster Size (*K*) and Bandwidth (BW)

For the local partitioning we have utilised KM and MS algorithms. The cluster size of the KM method and the Bandwidth (neighbour size) of the MS method largely control the performance of the clustering. In this subsection we investigate how these two parameters affect the overall performance which has been presented in Table 4. For this particular analysis we have only considered the 200x dataset and Model 1. We have utilised the values of *K* equal to 8, 16, and 24. As the value of *K* increases, the TP value also increases. This indicates that, with increasing *K*, the model performs in a specific way. Among the three values of *K* the best TN value (85.85%) is achieved when we utilise *K* = 8. Overall the best Accuracy is achieved when we utilise *K* = 8 which is slightly better than with *K* = 24.

For the MS method the obtained Precision values are 93.00%, 87.10%, and 89.00%, respectively, for BW equal to 0.2, 0.4, and 0.6, respectively. The best Accuracy performance (91.00%) is achieved when we utilise BW = 0.2. For both BW equal to 0.4 and 0.6 the obtained Accuracy was 87.00% which is less than when BW is equal to 0.2.

## 6. Recent Findings for Breast Image Classification Based on DNN

DNN methods have been implemented for breast image classification with some success.  Table 5 shows recent findings of breast cancer image classification based on the DNN method used for histopathological images (other than the BreakHis dataset). The best Accuracy performance of 92.45% is achieved by Bejnordi et al. [27].

However, we cannot exactly compare our performance with this existing finding because of the different datasets. We have compared our findings with the findings based on the BreakHis dataset which are presented in Table 6. Spanhol et al. classify the BreakHis dataset into benign and malignant classes using a CNN model and a few other models. Their CNN model is similar to the AlexNet CNN architecture and their finding (best one) has been listed in Table 6. In our experiment for the 40x dataset, we obtained 90.00% Accuracy whereas Spanhol et al. [28] obtained 90.40%. However, for the 100x, 200x, and 400x datasets the best achieved accuracies in our experiment are 90.00, 91.00, and 90.00%, respectively, which is better than the findings of Spanhol et al. [28]. Apart from this, Spanhol et al. [28] have no information about the sensitivity, Precision, recall, and MCC values. In this work we have explained those issues in detail. The original image of the BreakHis dataset is 760 × 460 × 3 pixels, and when Spanhol et al. [28] use this image they convert it to 350 × 230 × 3 pixels. However, we have utilised an image of 32 × 32 × 3 pixels which has reduced the computational latency [28]. Dimitropoulos et al. [29] utilised the Grassmannian Vector of Local Aggregated Descriptor (VLAD) method for the BreakHis dataset classification. Their finding is comparable to our finding. However, in their paper, they did not utilise the DNN models. Also, they do not describe the sensitivity, specificity,* F*-Measure, and MCC values, whereas we have explained those terms explicitly.

## 7. Conclusion

The judgement about benign and malignant status from digital histopathological images is subjective and might vary from specialist to specialist. CAD systems largely help in making an automated decision from the biomedical images and allow both the patient and doctors to have a second opinion. A conventional image classifier utilises hand-crafted local features from the images for the image classification. However, the recent state-of-the-art DNN model mostly employs global information using the benefit of kernel-based working techniques, which act to extract global features from the images for the classification. Using this DNN model, this paper has classified a set of breast cancer images (BreakHis dataset) into benign and malignant classes.

Images normally preserve some statistical and structural information. In this paper, to extract the hidden structural and statistical information, an unsupervised clustering operation has been done and the DNN models have been guided by this clustered information to classify the images into benign and malignant classes. At the classifier stage both Softmax and SVM layers have been utilised and the detailed performance has been analyzed. Experiments found that the proposed CNN-based model provides the best performance other than the LSTM model and the combination of LSTM and CNN models. We have found that, in most cases, Softmax layers do perform better than the SVM layer.

Most of the recent findings on the BreakHis dataset provide information about the Accuracy performance but do not provide information about the sensitivity, specificity, Recall,* F*-Measure, and MCC; however, we have explained these issues in detail. The best specificity, sensitivity, Recall, and* F*-Measure are 96.00%, 93.00%, 96.00%, and 93.00%, respectively. Of these issues, this paper has explained how the Accuracy, MCC, and loss values change with different epochs.

Providing a definite conclusion about the biomedical situation needs to be considered as it is directly related to the patient's life. In a practical scenario, the classification outcome of the BC images should be 100.00% accurate. Due to the complex nature of the data we have obtained 91% Accuracy, which is comparable with the most recent findings. There are a few avenues for obtaining more reliable solutions such as the following:

(i) Each histopathological image contains cell nuclei, which provide valuable information about the malignancy. So the DNN model guided by the cell nuclei orientation and position can improve the performance, since it provides more objective information to the network.
(ii) As our dataset is comparatively too small to be used with a DNN model, in the future the following two cases can be considered:
  (1) Data Augmentation
  (2) Transfer Learning
  with some fine local tuning.
(iii) Locally hand-crafted features also provide valuable information. So parallel feeding of the local data along with the raw pixels could improve the model's performance with reference to Accuracy.

## Figures and Tables

**Algorithm 1:**
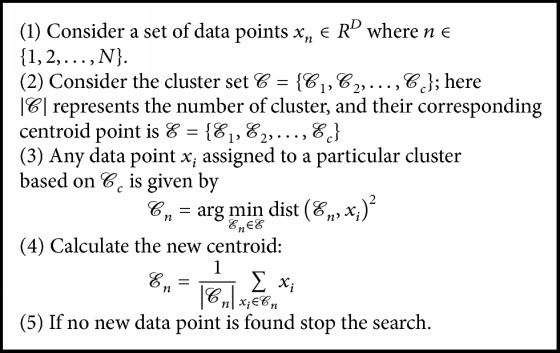
*K*-Means algorithm [18].

**Algorithm 2:**
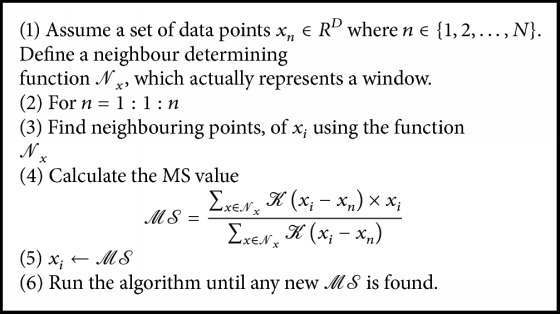
Mean shift algorithm [19].

**Table 1:** Cancer statistics for Australia 2017 [1].

	Female	Male	Total
Estimated number of new diagnoses (all cancers)	62005	72169	134174
Estimated number of deaths	20677	27076	47753
Estimated new cases of diagnosis (breast cancer)	17586	144	17730
Deaths due to breast cancer	3087	57	3114

**Table 2:** Comparison of TN, FP, FN, and TP values% for the different algorithms and different datasets.

Dataset(x)	Cluster	Decision	Model 1				Model 2				Model 3			
		Algorithm	TN	FP	FN	TP	TN	FP	FN	TP	TN	FP	FN	TP
40x	MS	SVM	68.39	31.60	8.00	92.00	53.55	46.44	5.20	94.76	59.10	40.00	5.00	95.00
		Softmax	93.00	7.00	19.00	81.00	75.28	24.71	12.23	87.76	68.39	31.60	9.40	90.50
	KM	SVM	67.00	32.00	6.00	93.10	53.00	46.99	7.00	92.00	70.68	29.30	10.00	90.00
		Softmax	84.82	15.51	7.00	92.94	72.98	27.01	10.58	89.51	70.68	29.31	6.90	93.10
	OI	SVM	62.00	37.00	5.00	94.00	66.00	34.00	10.00	90.00	72.00	28.00	11.00	89.00
		Softmax	77.00	23.00	6.00	93.00	74.00	26.00	10.00	90.00	78.00	21.00	5.00	94.00

100x	MS	SVM	68.00	32.00	11.00	88.00	66.00	33.00	9.00	90.11	53.00	46.00	7.00	92.00
		Softmax	80.00	20.00	6.00	94.00	72.57	27.42	17.00	82.00	54.85	45.14	10.00	90.00
	KM	SVM	80.00	2.00	6.00	94.00	56.00	44.00	6.10	93.80	66.00	34.00	12.00	88.00
		Softmax	75.00	25.00	4.00	95.96	64.71	35.42	12.00	87.20	61.14	38.87	10.10	89.00
	OI	SVM	70.00	30.00	8.00	92.00	56.00	44.00	4.00	96.00	71.00	28.00	6.00	93.00
		Softmax	64.00	36.00	8.00	92.00	71.00	29.00	12.00	88.00	73.00	26.00	8.00	91.00

200x	MS	SVM	70.70	29.00	4.10	95.82	61.61	38.38	4.00	95.30	72.00	27.00	6.80	93.00
		Softmax	81.00	19.00	5.00	95.00	75.75	24.24	9.80	90.17	65.00	35.00	2.00	97.00
	KM	SVM	69.69	30.30	3.60	96.00	64.14	35.85	5.00	94.00	75.00	24.00	8.00	91.00
		Softmax	85.85	14.16	8.00	91.00	78.00	21.00	12.00	87.00	71.71	22.20	6.00	93.00
	OI	SVM	73.00	27.00	4.00	96.00	63.63	36.66	6.00	93.00	76.00	23.00	6.00	94.00
		Softmax	78.00	22.00	5.00	94.10	76.00	24.00	12.00	88.00	70.00	30.00	6.00	93.00

400x	MS	SVM	68.30	31.69	4.00	95.31	53.55	46.44	5.20	94.76	61.20	38.79	5.70	94.21
		Softmax	84.00	15.00	6.00	93.00	65.01	34.97	9.30	90.06	61.20	38.79	6.00	93.38
	KM	SVM	68.00	31.00	4.00	95.00	53.00	46.99	7.00	92.00	59.01	40.98	6.61	93.38
		Softmax	78.00	22.00	4.00	96.00	63.93	36.06	11.57	88.42	65.00	35.00	5.00	95.00
	OI	SVM	75.00	25.00	6.00	94.00	61.00	39.00	12.00	88.00	79.95	24.06	9.00	90.00
		Softmax	76.00	24.00	10.00	90.94	70.00	30.00	10.00	90.00	82.51	17.48	12.67	87.32

**Table 3:** Average time and parameters for various TS and ID.

TS	ID	Average time (s)	Parameters
24	128	191	58280
32	96	240	52904
48	64	346	47528
64	48	438	44840
96	32	636	42152
128	24	822	40808

**Table 4:** Effect of the cluster size (*K*) and the bandwidth (BW) (%).

		TN	FP	FN	TP	Precision	*F*-Measure	Accuracy
KM	*K* = 8	85.85	14.16	8.00	91.00	91.00	92.00	90.00
	*K* = 16	77.00	23.00	06.00	94.00	90.00	92.00	88.90
	*K* = 24	77.00	23.00	05.00	95.00	90.00	93.00	89.75

MS	BW = 0.2	81.00	19.00	5.00	95.00	93.00	93.00	91.00
	BW = 0.4	70.00	30.00	04.00	96.00	87.10	91.00	87.00
	BW = 0.6	76.00	24.00	06.00	94.00	89.00	91.00	87.00

**Table 5:** CNN and histopathological findings.

Authors	Dataset	Method	Augmentation	Number of classes	Accuracy %	Sensitivity %	Recall %	ROC
Araujo et al. [16]	[26]	CNN	YES	2	80.60	70.00	—	—
Araujo et al. [16]	[26]	CNN + SVM	YES	2	83.20	80.00	—	—
B. Bejnordi	BREAST	CNN	YES	—	92.00	—	—	92.00
Bejnordi et al. [27]	[26]	CNN	YES	—	92.45	—	—	—

**Table 6:** Comparing Accuracy (%) in different models.

	40x	100x	200x	400x
CNN [28]	90.40	87.40	85.00	83.80
VLAD [29]	91.80	92.10	91.40	90.20
PFTAS [29]	83.80	82.10	85.10	82.30
ORB [29]	74.40	69.40	69.60	67.60
LPQ [29]	73.80	72.80	74.30	73.70
LBP [29]	75.60	73.20	72.90	73.10
GLCM [29]	74.70	78.60	83.40	81.70
CLBP [29]	77.40	76.40	70.20	81.80
